# Activities and Role Perceptions of Clinical Research Nurses in Participating Italian Scientific Research Hospitals: A Multicentre Cross-Sectional Survey

**DOI:** 10.3390/healthcare14183032

**Published:** 2026-09-16

**Authors:** Silvia Elettra Revere, Alberto Dal Molin, Gianluca Catania, Mattia Bozzetti, Daniele Napolitano, Davide Soranna, Gianfranco Parati, Daniele Privitera, on behalf of the CRN Italian Group

**Affiliations:** 1 Department of Cardiovascular, Neural and Metabolic Sciences, IRCCS Istituto Auxologico Italiano, San Luca Hospital, 20149 Milan, Italy; gianfranco.parati@unimib.it; 2 Department of Translational Medicine, Università del Piemonte Orientale, 28100 Novara, Italy; alberto.dalmolin@med.uniupo.it; 3 Department of Health Sciences, University of Genoa, 16132 Genoa, Italy; gianluca.catania@unige.it; 4 Department of Biomedicine and Prevention, University of Rome Tor Vergata, 00133 Rome, Italy; mattia.bozzetti@asst-cremona.it; 5 CEMAD—Fondazione Policlinico Gemelli, IRCCS, 00168 Rome, Italy; daniele.napolitano@policlinicogemelli.it; 6 Biostatistics Unit, IRCCS, Istituto Auxologico Italiano, 20149 Milan, Italy; d.soranna@auxologico.it; 7 Department of Health, Medicine and Life Sciences, Faculty of Science, Technology and Medicine, University of Luxembourg, L-4364 Esch-sur-Alzette, Luxembourg; daniele.privitera@uni.lu

**Keywords:** clinical research nurse, trials, clinical research, nursing role, professional identity

## Abstract

**Highlights:**

**What are the main findings?**

- Clinical research nurses rated all surveyed activities as highly important, but their reported involvement was uneven and concentrated on trial implementation and investigational medicinal product management rather than protocol assessment and planning.
- Workload-related stress and role ambiguity were reported by 48% and 39% of respondents, respectively, despite generally high perceived role adequacy and job satisfaction.

**What are the implications of the main findings?**

- Italian research hospitals should involve clinical research nurses earlier in protocol development and planning and define their competencies consistently across institutions.
- Structured education, career pathways and organisational support are needed to reduce role ambiguity and make fuller use of clinical research nurses’ expertise, potentially improving trial quality and workforce sustainability.

**Abstract:**

**Background/Objective:** Clinical research nurses (CRNs) support participant care and trial conduct, yet their role is inconsistently recognised and integrated in Italian Scientific Institutes for Research, Hospitalisation and Healthcare (IRCCSs). This study aimed to describe the perceived importance and self-reported frequency of CRN activities, professional-role perceptions and selected organisational characteristics among CRNs working in participating Italian IRCCSs. **Methods:** A cross-sectional online survey used the validated Italian Clinical Trials Nursing Questionnaire (149 items; 12 sections) via REDCap. All 53 IRCCSs were contacted. Eligible nurses worked in an IRCCS and devoted at least 50% of their working time to research-related nursing activities. Complete eligible questionnaires were summarised descriptively. **Results:** Twenty-five IRCCSs provided contacts and 308 CRNs were invited; 167 questionnaires were completed and 149 were eligible. Respondents had a median age of 38 years (IQR 31.5–48.5) and 4 years of CRN experience (IQR 2–7.5); 19% were male. Mean perceived importance was high across activity sections (4.06–4.65), while mean frequency varied (2.90–4.08). Protocol assessment (2.90) and planning (2.99) were least frequent; investigational medicinal product management (4.08) and implementation/evaluation (3.88) were more frequent. Workload-related and role-ambiguity stress were reported by 48% and 39%, respectively, despite high role adequacy (86%) and job satisfaction (81%). **Conclusions:** CRNs considered all assessed functions important, but their involvement was concentrated in trial delivery rather than upstream protocol work. Respondents with higher education reported more frequent involvement in protocol assessment/planning, recruitment, and informed consent, although these findings are descriptive.

## 1. Introduction

Over the past two decades, clinical research has evolved into an increasingly complex and multidisciplinary field, requiring the integration of diverse professional roles beyond the traditional physician–researcher model [1]. Among these, the Clinical Research Nurse (CRN) has emerged as a pivotal figure, contributing not only to patient care but also to the scientific and organisational aspects of clinical trials [2].

On the one hand, the role of the CRN is established and defined, particularly in countries such as the United Kingdom [3,4]. Across the European Union, the situation differs by country, reflecting national policies and health-system structures; in low- and middle-income settings, constrained funding and suboptimal working conditions substantially limit the availability and effectiveness of CRNs [5,6]. On the other hand, CRNs are recognised as essential members of the clinical research team, involved in protocol development, ethical oversight, data management, participant recruitment, and the coordination of trial procedures [3,4,7]. Their responsibilities encompass a wide range of tasks, including adverse event monitoring, informed consent processes, and ensuring compliance with Good Clinical Practice (GCP) guidelines [6].

Italian CRNs often operate with a strong focus on the technical and procedural aspects of care, such as managing investigational medicinal product (IMP) or performing trial-related assessments, while their involvement in managerial or organisational activities tends to be limited [8]. Moreover, the lack of a formal national framework—such as standardised job descriptions, a national registry, or defined core competencies—hinders the full professionalisation of the role [9].

Despite this, the presence of CRNs has been shown to significantly enhance the quality and continuity of care for research participants, thereby improving patient adherence, data integrity, and overall trial performance [10]. Furthermore, the reflexivity of nurses engaged in research activities—especially those in dual clinical-research roles—has increasingly been recognized as critical to the quality and ethical robustness of studies. This dual role demands a conscious negotiation between professional identity, research objectives, and patient-centred care [11]. To our knowledge, no dedicated national register or census of CRNs is available in Italy; therefore, the size of the Italian CRN workforce cannot be reliably estimated. Professional registration as a nurse represents the baseline requirement, whereas no uniform CRN-specific postgraduate qualification or career pathway has been established. European pathways are similarly heterogeneous: in a survey involving 38 countries, a nursing degree was reported as necessary in 33 countries, GCP training in 24, and dedicated research-nurse courses were available in 18 [6].

However, current Italian evidence remains limited. The previous Italian CTNQ survey included only 30 nurses, was conducted more than a decade ago within oncology settings, and described a predominantly task-oriented role, with limited involvement in protocol assessment, planning, recruitment, and data management [8]. Although the need for clearer role recognition [9] and systematic assessment of trial-site performance [12] has been identified, it remains unclear whether this activity profile persists across different specialties and research designs within IRCCSs, which combine specialised care and research [13]. Available single-centre evidence cannot characterise CRN practice across institutions [14]. Therefore, a multicentre assessment of CRN activities, role perceptions, and organisational characteristics is needed to inform role definition, education, and organisational support.

This study aimed to describe the perceived importance and self-reported frequency of CRN activities, professional-role perceptions and selected organisational characteristics among CRNs working in participating Italian IRCCSs. A secondary descriptive objective was to summarise CTNQ domain scores according to participants’ highest nursing qualification.

## 2. Materials and Methods

### 2.1. Study Design

This study was a multicenter cross-sectional online survey aimed at delineating the role of CRNs in Italy. The design was informed by a previous national survey conducted in Italian oncology hospitals [8] and follows the Consensus-Based Checklist for Reporting of Survey Studies (CROSS) guidelines [15].

### 2.2. Sampling and Recruitment

All 53 Italian IRCCSs were contacted via email, with up to three follow-ups. We reached out to the scientific departments and, where applicable, the healthcare professions departments. Institutions agreeing to participate identified a contact person responsible for providing the names and institutional email addresses of eligible CRNs. The inclusion criteria required nurses to be employed at an IRCCS and to dedicate at least 50% of their working time to research-related nursing activities, encompassing both pharmacological and non-pharmacological studies. The ≥50% threshold was an investigator-defined operational criterion intended to identify nurses whose principal occupational allocation involved clinical research. Eligible nurses received a personalised email with study details and the survey link. A follow-up email was sent after two weeks to encourage participation. In addition, all CRNs were offered the opportunity to be recognised as collaborative authors of the study.

### 2.3. Sample Characteristics

Participants were CRNs working within Italian IRCCSs and actively engaged in clinical research encompassing both patient care and protocol management. Eligibility was confirmed through screening questions at the beginning of the survey. Sociodemographic information, organizational setting, and professional background were collected in the final section of the questionnaire. Only fully completed responses meeting the eligibility criteria were included in the final analysis.

### 2.4. Study Instrument

The instrument used was the Italian validated version of the Clinical Trials Nursing Questionnaire (CTNQ), developed to assess the frequency and perceived importance of activities performed by CRNs. The internal consistency of the questionnaire was evaluated using the Cronbach’s alpha coefficient, which was found to be 0.98 for the frequency scale and 0.96 for the importance scale [16]. The CTNQ includes 149 items across 12 sections, grouped into five thematic areas: clinical trial activities (Sections 1–8), role perception (Section 9), nursing role characteristics (Section 10), organizational context (Section 11), and demographic information (Section 12). Sections 1–8 use two separate five-point Likert scales (1–5) to assess activity frequency and importance. The CTNQ was created for nurses involved in clinical trials. In this study, participants were also able to indicate their involvement in observational and other non-trial research designs. Therefore, Section 5, which pertains to IMP, was completed only by those respondents who confirmed their current or past involvement in pharmacological studies. Responses marked as “not applicable” were excluded from the item-specific denominators and from the calculation of domain scores. Questions related to study design and to nurse-led research allowed multiple responses. Items on postgraduate course type and career opportunities also permitted multiple answers.

### 2.5. Data Collection

The survey was self-administered, anonymous, and hosted on REDCap, a secure web-based platform. The invitation email included all relevant information, and the system was configured to allow only one response per participant. No incentives were offered. At the end of the survey, participants could optionally express interest in joining a study-related working group. All responses were automatically recorded in REDCap and exported for analysis without any identifying information, ensuring participant confidentiality and data security throughout the process.

### 2.6. Ethical Consideration

This was a non-interventional cross-sectional survey involving adult healthcare professionals only. No patients, clinical interventions, biological materials, patient records, or patient health data were involved. The protocol and data-processing arrangements were reviewed by the Data Protection Officer of Fondazione IRCCS Istituto Auxologico Italiano. Participants received information regarding the study purpose, voluntary participation, confidentiality, data use, and their right to discontinue the questionnaire before submission. According to applicable institutional policies for anonymous questionnaire studies, a formal ethics committee was not required. Electronic informed consent for participation was obtained from all participants before access to the questionnaire.

### 2.7. Statistical Analysis

Analyses were planned as descriptive. Only fully completed questionnaires meeting eligibility criteria were included (complete-case analysis). Participant characteristics and organisational variables were summarised as counts and percentages for categorical data, and as medians with interquartile ranges (IQRs) for continuous variables. For the CTNQ, items are rated on 5-point Likert scales for frequency and perceived importance. For each CTNQ section (Sections 1–8), section-level frequency and importance scores were computed by averaging item responses within the same section and are reported as mean with standard deviation (SD) and median. Section 5 (IMP–related activities) was administered only to respondents who reported current or previous involvement in pharmacological trials and was analysed within that subgroup. Section 9, concerning role perception, was assessed using a 5-point Likert scale (1 = strongly disagree–5 = strongly agree).

For graphical synthesis, item/section scores were collapsed into three categories to facilitate interpretation: for frequency, “never performed” (=1), “low-frequency performed” (>1 to <4), and “high-frequency performed” (≥4 to 5). For importance, “not important” (=1), “less important” (>1 to <4), and “more important” (≥4 to 5). Descriptive stratified summaries of CTNQ section scores were produced according to education level (≤Bachelor with/without postgraduate courses vs. ≥Master’s/PhD), reporting mean and median values by stratum. Statistical analyses were performed using SAS statistical software (version 9.4; SAS Institute).

## 3. Results

### 3.1. Respondent Characteristics

**Figure 1:**
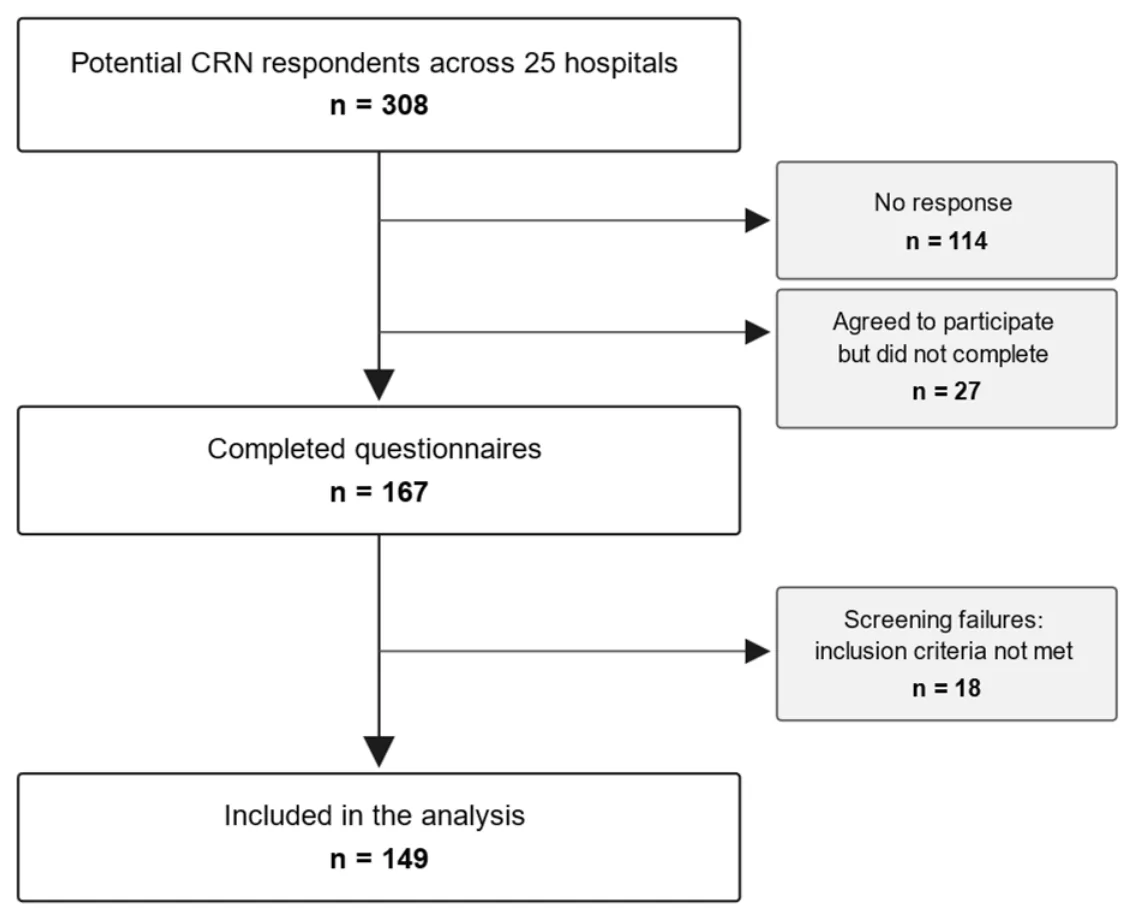
Flow diagram of survey response, eligibility screening, and inclusion in the final analysis.

All 53 Italian IRCCS hospitals were invited to participate. Of these, 25 (47%) provided a designated contact and received the online survey; one hospital reported having no CRN service. Overall, the survey was distributed to 308 potential CRN respondents across the 25 hospitals; of these, 114 never responded, and 27 CRNs agreed to participate but didn’t complete the questionnaire. A total of 167 questionnaires were completed; 18 were identified as screening failures for not meeting the inclusion criteria, leaving 149 for analysis (Figure 1). Participants were predominantly female, with 29 males (19%). The median age was 38 years (IQR, 31.5–48.5). Respondents were mainly from North-West Italy (48%). Regarding nurses’ education, 54% held a bachelor’s degree in nursing and 23% a master’s degree in nursing sciences, while only 5% had a PhD. The median number of years in nursing was 14 (IQR, 8–25), with 33% of respondents reporting 21 years or more. Median years in clinical nursing were 10 (IQR, 4.5–16.5) and the median years in clinical research nursing were 4 (IQR, 2–7.5). Most respondents reported involvement in randomised controlled trials with substantial participation also in observational designs; there was less involvement in qualitative studies. The clinical research areas most frequently reported were oncology (37%), cardiology (19%), and neurology (15%). The remaining information can be found in Table 1.

### 3.2. Evaluation of the Activities and Responsibilities (Sections 1 and 8)

**Figure 2:**
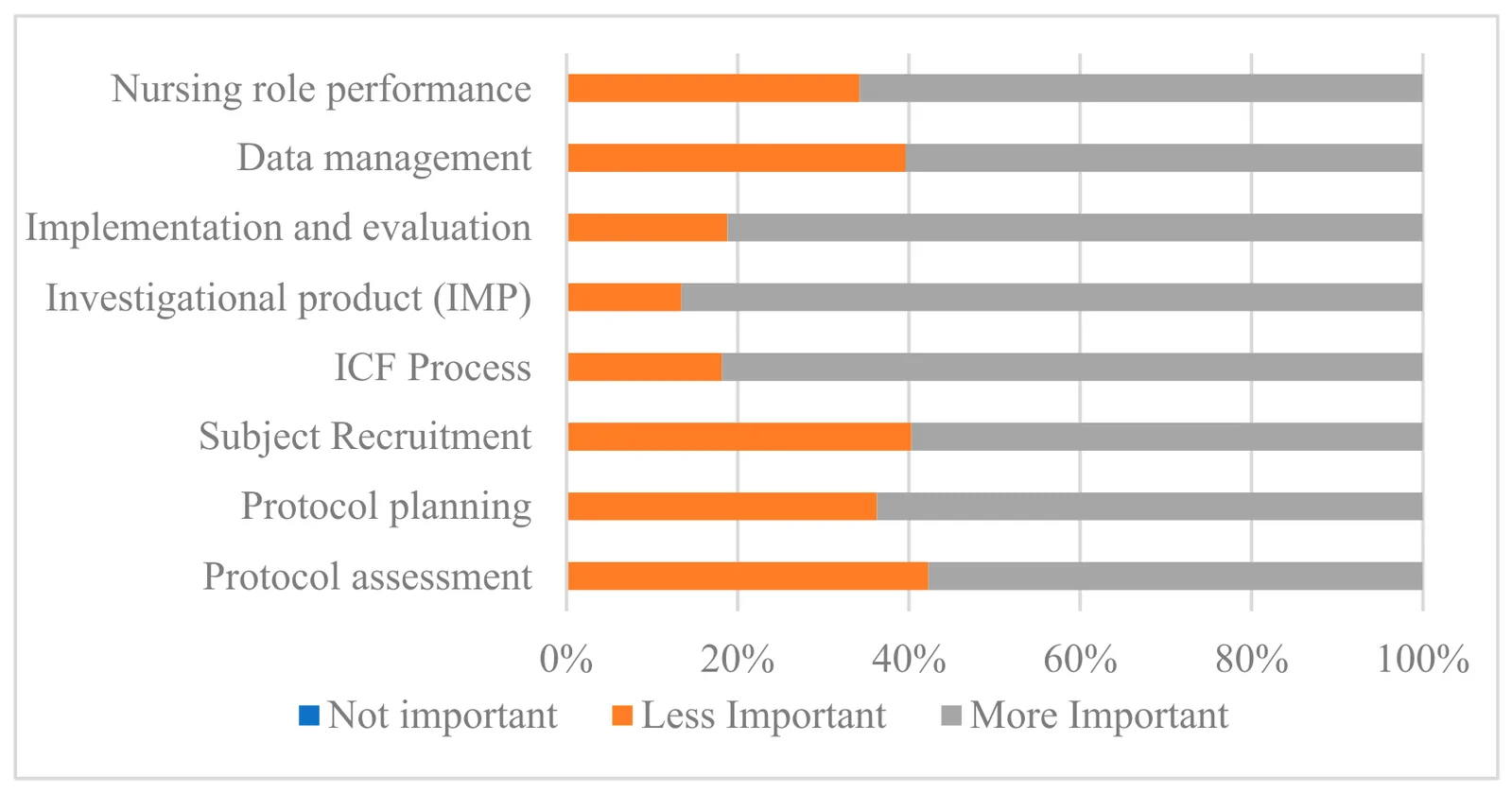
Stratification of activity importance. Item- and section-level scores were collapsed into three categories to facilitate interpretation: not important (score = 1), less important (score > 1 and <4), and more important (score ≥ 4 and ≤5). The IMP domain includes 119 eligible respondents; all other domains include 149 respondents. Note: ICF: Informed Consent Form.

**Figure 3:**
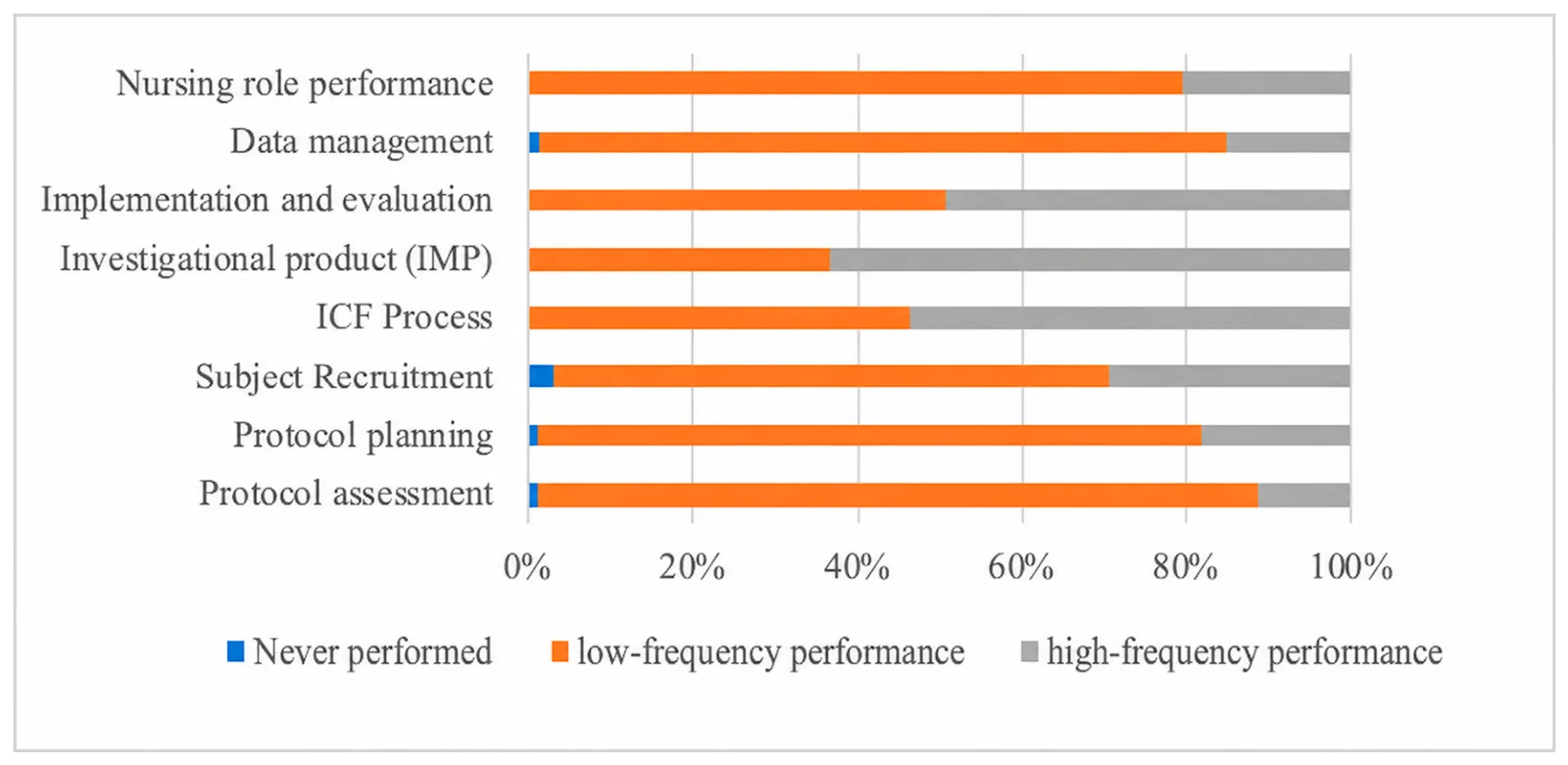
Stratification of activity frequency. Item- and section-level scores were collapsed into three categories to facilitate interpretation: never performed (score = 1), low-frequency performed (score > 1 and <4), and high-frequency performed (score ≥ 4 and ≤5). Note: ICF: Informed Consent Form.

Section-level CTNQ scores showed a consistent pattern of high perceived importance across all domains (mean importance range 4.06–4.65), suggesting that respondents view core clinical research nursing activities as broadly relevant to safe and effective research conduct (Table 2 and Figure 2). In contrast, mean frequency scores ranged from 2.90 to 4.08, and the lowest reported frequencies were observed for upstream tasks such as protocol assessment (Section 1; mean 2.90) and protocol planning (Section 2; mean 2.99). Operational trial conduct activities were reported more frequently, particularly IMP management (Section 5; mean 4.08) and implementation/evaluation (Section 6; mean 3.88). The IMP domain received responses from 119 participants who reported current or previous involvement in pharmacological trials. Since this domain used a different denominator, its estimates should not be directly compared to those from domains based on all 149 respondents. Informed consent activities were among the most valued (Section 4; mean importance 4.54) and were performed relatively often (mean frequency 3.80) (Table 2 and Figure 3). The lowest- and highest-rated items (mean scores) for frequency and importance across CTNQ sections are presented in Table 3. In the unadjusted descriptive stratification, respondents with a Master’s degree or PhD (*n* = 41) reported higher mean frequency scores for protocol assessment, protocol planning, subject recruitment and informed-consent activities than respondents with a Bachelor’s degree or lower, with or without postgraduate courses (*n* = 108) (Tables S1 and S2). No inferential testing or adjustment for experience, organisational role, institution or study portfolio was performed.

### 3.3. Evaluation of Perception of the Professional Nursing Role (Section 9)

**Figure 4:**
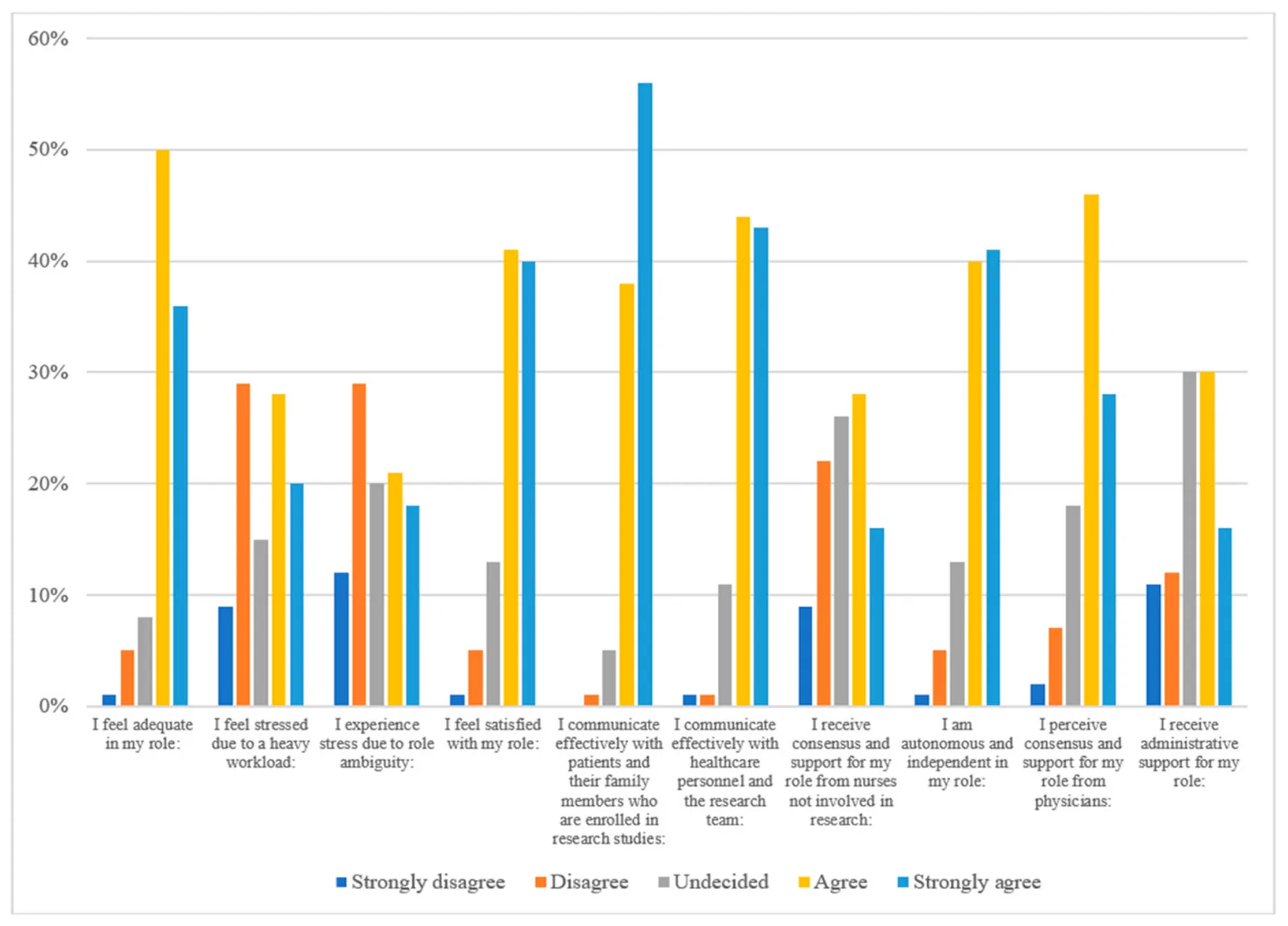
Nursing role perception among Italian CRNs.

As shown in Figure 4, most respondents reported feeling adequate in their role (86% agreement/strong agreement). Nearly half (48% agreement/strong agreement) reported workload-related stress, while 38% disagreed and 15% were undecided. Role ambiguity-related stress was reported by 39% (agreement/strong agreement), with 41% disagreeing and 20% undecided. Role satisfaction was high (81% agreement/strong agreement). Communication was rated positively by both patients/families (94% agreement/strong agreement) and healthcare/research teams (87% agreement/strong agreement). Perceived support was higher from physicians (74% agreement/strong agreement) than from non-research nurses (44%) and administrative staff (46%).

### 3.4. Nursing Role and Organisational Characteristics

As illustrated in Table 4, engagement in formal trial leadership roles among this cohort of CRNs was limited. Specifically, 55% reported never having acted as a study coordinator, while 35% indicated they had *done* so at least occasionally. Additionally, 76% of respondents indicated that they had never served as a Principal Investigator (PI). The survey did not specify who held PI or governance responsibilities, making it impossible to determine how these responsibilities were distributed among professional groups. Additionally, 76% of respondents indicated that they had never served as a Principal Investigator (PI). The survey did not specify who held PI or governance responsibilities, making it impossible to determine how these responsibilities were distributed among professional groups. Employment arrangements for CRNs were largely stable, with 98% of respondents reporting full-time employment. Most participants (76%) reported having a formal job description. However, perceived opportunities for professional advancement varied: 25% said they had such opportunities, 38% said they did not, and 37% were unsure. Among the opportunities reported, 23% of respondents attended conferences, 17% received training support, and only 8% mentioned opportunities for salary progression.

## 4. Discussion

In this multicenter cross-sectional survey of CRNs working in Italian IRCCSs, perceived importance was consistently high across clinical nursing activities, while reported frequency varied across domains. Respondents considered all nursing functions highly important, whereas the reported frequency of performance differed across activities in daily practice. The lowest reported frequencies concerned upstream activities, specifically protocol assessment and protocol planning. In contrast, operational and delivery tasks—such as IMP management and implementation/evaluation—were performed more frequently. Activities related to informed consent were both highly valued and performed relatively frequently, reflecting the critical role of CRNs in ensuring ethical conduct and supporting participants.

The activity profile observed here aligns closely with previous Italian evidence derived from the CTNQ [8]. In particular, the national oncology nursing survey by Catania and colleagues reported a role heavily weighted toward trial delivery and procedural tasks, with reduced involvement in protocol development and other upstream functions [8]. Our findings extend this pattern beyond oncology to the entire IRCCS network, indicating that underutilization in upstream phases may be a structural feature of site organization rather than a specialty-specific phenomenon. Similar findings are reported in another national survey from Sweden, which indicated that the tasks most frequently performed and considered the most important by the CRNs involved informed consent and the management of IMP [17]. However, a lower CRN involvement in budgetary, regulatory, data-management and feasibility activities may reflect task allocation to other research professionals rather than underutilisation; however, staffing and delegation models were not assessed.

In this context, a lower frequency of protocol assessment and planning may indicate an incomplete nursing realisation of the study management and scientific contribution aspects, even though their perceived importance is high. This configuration is compatible with an organisational model in which CRNs are primarily positioned as trial-delivery specialists, while feasibility appraisal, protocol shaping, and scientific decision-making are concentrated within physician leadership or sponsor-driven processes [18].

Overall, the profile emerging from our sample depicts the CRN as closer to what the literature commonly describes as a research nurse/clinical research nurse focused on research delivery (ethical and effective trial conduct, protocol adherence, participant support, and day-to-day study coordination) than to the nurse researcher, whose core remit is the generation of new knowledge through the formulation of research questions, study design, rigorous conduct, data analysis, and dissemination [19]. In addition, the educational profile observed in our study (23% holding a Master of Science and only 5% a PhD) is compatible with this role configuration, since the PhD is widely characterised as the research-focused qualification that prepares nurses to create, translate, and communicate new knowledge as scientific leaders, and is therefore strongly associated with sustained nurse-scientist trajectories [19,20].

Scope-and-standards documents for clinical research nursing explicitly frame CRN practice as encompassing engagement with protocol-relevant processes, quality systems, and ethical safeguards; therefore, restricted upstream participation may represent a mismatch between professional standards and local role allocation [21].

These descriptive results are compatible with several unmeasured organisational factors, including local delegation pathways, accountability structures, staffing and protected time. These factors are possible explanations and were not directly evaluated in the present survey. The prominent position of informed consent in this survey is consistent with its centrality within the human-subject protection dimension of clinical research nursing. Contemporary ethics and regulatory guidance increasingly treat informed consent as an ongoing communication and support process rather than a single administrative event [18].

CRNs’ longitudinal contact with participants and proximity to bedside care uniquely position them to detect misunderstanding, support deliberation, and reinforce voluntariness, thereby strengthening both participant experience and protocol adherence. Accordingly, safeguarding CRN capacity for consent-related work—through adequate staffing and clear responsibility boundaries—should be considered an ethical and quality priority [22].

In the unadjusted descriptive stratification, respondents with a Master’s degree or PhD reported higher mean frequency scores for selected upstream activities, including participant recruitment and informed consent, than the comparison group, while importance ratings remained consistently high across educational levels. Although this pattern is compatible with competency-based models, according to which autonomy and scope of responsibility develop through structured learning, supervised progression, and demonstrated competence rather than through years of experience alone, it should not be interpreted as evidence of an educational effect. No inferential testing was conducted, and the analyses were not adjusted for years of experience, organisational role, institution, or study portfolio. Furthermore, the survey did not assess the content or duration of research-specific training. Consequently, the observed differences cannot be attributed to educational attainment and should not be interpreted as demonstrating differences in competence [23]. However, it is important to note that there is considerable variability in the courses and training offered to research nurses, both in terms of duration and the diversity of the educational training [6].

In addition, the combination of high job satisfaction alongside significant workload-related and ambiguity-related stress suggests that work is seen as meaningful and professionally valued, but might still be limited by organisational conditions. For example, role ambiguity is a recognized factor that contributes to strain and burnout in healthcare settings [9,24]. This ambiguity can stem from inconsistent job boundaries, unclear task delegation, and a lack of proper integration of CRNs within both clinical units and administrative structures [6]. This makes it challenging to justify adequate staffing and protected time without using trial-specific workload measurement tools. Evidence from oncology research programs shows that structured workload assessment can guide staffing decisions and enhance the stability of trial delivery [25]. Therefore, in the IRCCS context, a systematic evaluation of workload could promote both quality care and workforce well-being, shifting staffing justifications from anecdotal to data-driven planning [12].

These findings support the need for a coordinated approach to CRN role definition in Italy, centred on standardized job descriptions, core competencies, and minimum expectations for involvement across the trial lifecycle. Priorities include formalizing CRN participation in feasibility assessment and protocol planning, adopting workload-based staffing approaches, and providing protected time for training and quality activities [6,26]. Earlier CRN input into feasibility assessment and protocol planning could be evaluated prospectively, where consistent with local governance and role allocation. Potential effects on feasibility, protocol deviations, participant-centred implementation and workforce outcomes remain hypotheses for future research [5,27]. Finally, sustainability will require both recognition (career pathways, advancement options) and infrastructure (staffing models, mentorship), rather than reliance on individual motivation [10,28]. A strength of this study is the use of the validated Italian CTNQ and the inclusion of respondents from 25 IRCCSs working across different specialties and study designs. Nevertheless, the multicentre scope should not be interpreted as national representativeness.

This study has several limitations. First, 25 of the 53 IRCCSs approached contributed participant contacts, and almost half of the analysed respondents were based in North-West Italy. Institutions with more established CRN services may have been more likely to participate and identify eligible nurses, while individual participation was voluntary; the findings are therefore not nationally representative. Second, the ≥50% research-time eligibility criterion excluded nurses in mixed clinical–research roles and narrowed the population represented. Third, respondents were clustered within 25 institutions, but centre-level organisational variables and institutional clustering were not evaluated; high-enrolling centres may therefore have disproportionately influenced aggregate estimates. Fourth, exclusion of the 27 individuals who initiated but did not complete the survey may have introduced completion-related selection bias. Fifth, the cross-sectional self-reported data are susceptible to recall and social-desirability bias and provide no objective measures of workload, competence, role performance or research quality [29]. Sixth, the CTNQ was developed for clinical-trial nursing, whereas respondents reported working across heterogeneous study designs. Some activities may therefore have been non-applicable rather than infrequently performed. The IMP domain was restricted to 119 respondents and is not directly comparable with domains based on the full sample. Finally, staffing, delegation, study portfolio and governance arrangements were not measured. Consequently, the study cannot determine whether infrequently reported activities reflected limited CRN involvement, appropriate multidisciplinary task allocation or non-applicability. Education-stratified summaries were unadjusted and cannot support causal or competence-related conclusions.

Future research should therefore combine survey approaches with centre-level structural variables (e.g., trial volume, presence of trial offices, delegation models) and objective performance indicators (e.g., recruitment efficiency, protocol deviations, query rates), potentially using mixed methods to link role allocation to measurable quality and efficiency outcomes.

## 5. Conclusions

This multicenter cross-sectional survey of CRNs working in Italian IRCCS hospitals documents consistently high perceived importance across clinical research nursing activities, alongside more heterogeneous reported frequencies across domains. The lowest reported frequencies concerned upstream work (protocol assessment and protocol planning), even though respondents rated these activities as highly relevant within the clinical trial lifecycle.

In contrast, operational trial-conduct functions, particularly IMP management and implementation/evaluation, were performed more frequently. However, this domain was relevant only to 119 respondents who reported current or prior involvement in pharmacological trials. Informed consent activities were highly valued and frequently conducted, confirming their importance within CRN practice as reported by the respondents. This pattern seems to be compatible with an organisational configuration in which CRNs are positioned primarily as trial-delivery specialists, while feasibility appraisal and protocol-shaping responsibilities remain concentrated elsewhere in the governance chain. In addition, descriptive stratification seems to suggest that post-basic education is associated with a higher frequency of upstream activities as well as recruitment and informed consent work, while importance ratings remain high across educational groups.

## Figures and Tables

**Table 1:** Characteristics of the study sample of Clinical Research Nurses in Italy.

	All Cohort (*N* = 149)
**Male** *N* (%)	29 (19%)
**Age (years)** *median [IQR]*	38 [31.5–48.5]
**Geographic area (Italy)** *N* (%)	
Centre	42 (28%)
Islands	3 (2%)
North-east	25 (17%)
North-west	72 (48%)
South	7 (5%)
**Nursing Education** *N* (%)	
Diploma in Nursing	27 (18%)
Bachelor in Nursing	81 (54%)
Master of Science in Nursing	34 (23%)
Ph.D.	7 (5%)
**Postgraduate courses**	
Clinical Research Nursing	48 (32%)
Others	53 (36%)
**Years in nursing** *median [IQR]*	14 [8–25]
**Years in nursing (range)** *N* (%)	
0–5	18 (12%)
6–10	40 (27%)
11–20	42 (29%)
≥21	49 (33%)
**Years in clinical nursing** *median [IQR]*	10 [4.5–16.5]
**Years in clinical nursing** (range) *N* (%)	
0–5	43 (29%)
6–10	36 (24%)
11–20	39 (26%)
≥21	31 (20%)
**Years in clinical research** *median [IQR]*	4 [2–7.5]
**Years in clinical research** (range) *N* (%)	
0–1	31 (21%)
2–5	61 (41%)
6–10	39 (26%)
≥11	18 (11%)
**Employment status** *N* (%)	
Full-time	146 (98%)
Part-time	2 (1%)
Other	1 (1%)
**Types of studies involving CRNs** *N*	
Experimental studies	286
Analytical observational studies	179
Descriptive observational studies	125
Qualitative studies	29
Mixed methods study	12
Secondary research studies	26
***Trial Area*** *N (%)*	
Oncology	55 (37%)
Cardiology	29 (19%)
Neurology	23 (15%)
Genetics	20 (13%)
Gastroenterology	13 (9%)
Pediatrics	12 (8%)
Diagnostic imaging	11 (7%)
Infectious disease	6 (4%)
Psychiatry	4 (3%)
Dermatology	3 (2%)
Complex care medicine	3 (2%)
Rehabilitation	3 (2%)
Neurorehabilitation	2 (1%)
Ophthalmology	0 (0%)
Orthopedics	0 (0%)

Note: IQR: Interquartile Range.

**Table 2:** Frequency and importance ratings (on a scale of 1 to 5) for activities among Italian Clinical Research Nurses (sections 1–8). Note: ICF: Informed Consent Form; CTNQ: Clinical Trial Nurse Questionnaire; SD: Standard Deviation.

CTNQ Sections (149 Subjects)	Frequency		Importance	
	Mean [SD]	Median	Mean [SD]	Median
Protocol assessment	2.90 [0.94]	3.03	4.06 [0.78]	4.15
Protocol planning	2.99 [0.99]	2.94	4.15 [0.78]	4.19
Subject recruitment	3.20 [1.10]	3.21	4.07 [0.86]	4.08
ICF process	3.80 [1.03]	3.80	4.54 [0.60]	4.54
Investigational medicinal product (119 subjects)	4.08 [0.84]	4.20	4.65 [0.55]	4.73
Implementation and evaluation	3.88 [0.81]	3.94	4.52 [0.58]	4.55
Data management	3.01 [0.97]	3.19	4.13 [0.80]	4.20
Nursing role performance	3.21 [0.92]	3.27	4.30 [0.75]	4.30

**Table 3:** Lowest- and highest-rated items (mean scores) for frequency and importance across CTNQ sections (on a scale of 1 to 5).

CTNQ Sections	Lowest vs. Highest Scored Item (Mean)	
	Frequency	Importance
Protocol assessment	Negotiate the study budget (1.61) vs. Determine the availability of equipment and materials (3.84)	Negotiate the study budget (3.34) vs. Assess the protocol’s ability to ensure the patient’s rights, safety and well-being (4.55)
Protocol planning	Respond to requests for additional information from the Ethics Committee after consulting with the study sponsor (2.02) vs. Attend the study initiation visit (4.21)	Prepare the regulatory documents for the study sponsor (3.67) vs. Attend the study initiation visit (4.65)
Subject recruitment	Develop recruitment materials for the study (e.g., upload active protocols to the hospital website) (2.20) vs. Maintain records of patient screening (3.85)	Develop recruitment materials for the study (e.g., upload active protocols to the hospital website) (3.63) vs. Ensure that all required enrollment and eligibility procedures are complete (4.43)
ICF process	Verify that the written informed consent contains the essential elements of informed consent (3.35) vs. Provide support to the patient for any issues related to participation in the study (4.36)	Assess the potential patient’s goals and motivations for participating in the trial (4.34) vs. Verify that written informed consent is obtained before the patient participates in the study (4.76)
Investigational medicinal product	Order/procure the IMP (3.04) vs. Verify the dose of the investigational drug to be administered (4.64)	Order/procure the IMP (4.18) vs. Verify the dose of the investigational drug to be administered (4.86)
Implementation and evaluation	Report the serious adverse events required by the sponsor in accordance with regulations (3.14) vs. Ensure the proper collection, handling, and shipment of samples (4.59)	Conduct a psychosocial assessment of the patient and their family (4.16) vs. Ensure the proper collection, handling, and shipment of samples (4.77)
Data management	Prepare a written summary of the study status for the Ethics Committee (1.87) vs. Ensure the protection of patient data in accordance with regulations (4.22)	Prepare a written summary of the study status for the Ethics Committee (3.54) vs. Ensure the protection of patient data in accordance with regulations (4.61)
Nursing role performance	Participate in educational programs that inform the public and/or other healthcare professionals about cancer research and clinical trials (2.50) vs. Receive up-to-date information on the investigational drug regarding its administration, side effects, and patient monitoring (4.09)	Participate in educational programs that inform the public and/or other healthcare professionals about cancer research and clinical trials (4.09) vs. Receive up-to-date information on the investigational drug regarding its administration, side effects, and patient monitoring (4.53)

Note: ICF: Informed Consent Form; CTNQ: Clinical Trial Nurse Questionnaire; IMP: Investigational medicinal product.

**Table 4:** Organisational characteristics and research involvement of Clinical Research Nurses.

		All Cohort (*N* = 149)
		*N, %*
**Job Description**		
Yes		112 (76%)
No		23 (16%)
Unknown		14 (9%)
**Study coordinator** *N* (%)		
Never		83 (56%)
Rarely		14 (9%)
Sometimes		22 (15%)
Often		16 (11%)
Always		14 (9%)
**Principal Investigator** *N* (%)		
Never		113 (76%)
Rarely		18 (12%)
Sometimes		15 (10%)
Often		1 (1%)
Always		2 (1%)
**Authorship** *N* (%)		
Never		67 (45%)
Rarely		32 (21%)
Sometimes		28 (19%)
Often		18 (12%)
Always		4 (3%)
**Career opportunities**		
No		57 (38%)
Yes		37 (25%)
Don’t know		55 (37%)
If yes:	Pay rise	12 (8%)
	Conference attendance	35 (23%)
	Training support (course fees + paid study leave)	26 (17%)

## Data Availability

The data that support the findings of this study are available from the corresponding author upon reasonable request.

## Supplementary Materials

The following supporting information can be downloaded at https://www.mdpi.com/article/10.3390/healthcare14183032/s1. Table S1: Frequency rating (on a scale of 1 to 5) for activities among Italian Clinical Research Nurses (sections 1–8), stratified for nurse education level. Table S2: Importance rating (on a scale of 1 to 5) for activities among Italian CRNs (sections 1–8), stratified for nurse education level.

## Abbreviations

CRN	Clinical Research Nurse
CROSS	Consensus-Based Checklist for Reporting of Survey Studies
CTNQ	Clinical Trials Nursing Questionnaire
GCP	Good Clinical Practice
ICF	Informed Consent Form
IMP	Investigational Medicinal Product
IQR	Interquartile Range
IRCCS	Istituto di Ricovero e Cura a Carattere Scientifico (Scientific Institute for Research, Hospitalization and Healthcare)
