# Supplementary material for: Activities and Role Perceptions of Clinical Research Nurses in Participating Italian Scientific Research Hospitals: A Multicentre Cross-Sectional Survey
Source: Healthcare (Basel). 2026 Sep 16;14(18):3032. doi: 10.3390/healthcare14183032 (PMC13606684; doi:10.3390/healthcare14183032)
Supplement: Supplementary file 1 [file healthcare-14-03032-s001.zip › healthcare-4547085-supplementary.pdf]

SUPPLEMENTARY MATERIAL

Table S1: Frequency rating (on a scale of 1 to 5) for activities among Italian Clinical Research Nurses (sections 1-8), stratified for nurse education level.

CTNQ sections (149 subjects)	≤ Bachelor (plus postgraduate courses)		≥ Master's (MSc/PhD)	
	Mean	*Median*	*Mean*	*Median*
**Protocol assessment**	2.84	2.93	3.03	3.13
**Protocol planning**	2.95	2.92	3.16	3.21
**Subject recruitment**	3.08	3.23	3.43	3.53
**ICF process**	3.73	4.07	3.91	4.14
**Investigational medicinal product (119 subjects)**	4.08	4.35	4.04	4.2
**Implementation and evaluation**	3.95	4.04	3.65	3.73
**Data management**	2.94	2.83	3.20	3.22
**Nursing role performance**	3.19	3.2	3.21	3.3

Note: ICF: Informed Consent Form; CTNQ: Clinical Trial Nurse Questionnaire; MSc: Master of Science

PhD: Doctor of Philosophy

Table S2: Importance rating (on a scale of 1 to 5) for activities among Italian CRNs (sections 1-8), stratified for nurse education level.

**CTNQ sections (149 subjects)**	**≤ Bachelor (plus postgraduate courses)**		**≥ Master's (MSc/PhD)**	
	Mean	***Median***	***Mean***	***Median***
**Protocol assessment**	4.07	4.2	3.98	4.13
**Protocol planning**	4.16	4.28	4.07	4.28
**Subject recruitment**	4.09	4.30	3.93	3.84
**ICF process**	4.55	4.92	4.46	4.64
**Investigational medicinal product (119 subjects)**	4.61	4.95	4.75	4.9
**Implementation and evaluation**	4.58	4.86	4.29	4.39
**Data management**	4.15	4.33	4.06	4.11
**Nursing role performance**	4.33	4.7	4.19	4.4

Note: ICF: ICF: Informed Consent Form; CTNQ: Clinical Trial Nurse Questionnaire; MSc: Master of Science

PhD: Doctor of Philosophy
